# Inflammation as common link to progressive neurological diseases

**DOI:** 10.1007/s00204-023-03628-8

**Published:** 2023-11-15

**Authors:** Ana Dias-Carvalho, Susana Isabel Sá, Félix Carvalho, Eduarda Fernandes, Vera Marisa Costa

**Affiliations:** 1 Associate Laboratory i4HB - Institute for Health and Bioeconomy, Faculty of Pharmacy, University of Porto, 4050-313 Porto, Portugal; 2 UCIBIO- Applied Molecular Biosciences Unit, Laboratory of Toxicology, Department of Biological Sciences, Faculty of Pharmacy, University of Porto, 4050‐313 Porto, Portugal; 3 Unit of Anatomy, Department of Biomedicine, Faculty of Medicine, University of Porto, Porto, Portugal; 4 CINTESIS@RISE, Faculty of Medicine, University of Porto, Porto, Portugal; 5 LAQV, REQUIMTE, Laboratory of Applied Chemistry, Department of Chemical Sciences, Faculty of Pharmacy, University of Porto, 4050-313 Porto, Portugal

**Keywords:** Neurodegeneration, Central nervous system, Cytokines, Immune response, Neuroinflammation

## Abstract

Life expectancy has increased immensely over the past decades, bringing new challenges to the health systems as advanced age increases the predisposition for many diseases. One of those is the burden of neurologic disorders. While many hypotheses have been placed to explain aging mechanisms, it has been widely accepted that the increasing pro-inflammatory status with advanced age or “inflammaging” is a main determinant of biological aging. Furthermore, *inflammaging* is at the cornerstone of many age-related diseases and its involvement in neurologic disorders is an exciting hypothesis. Indeed, aging and neurologic disorders development in the elderly seem to share some basic pathways that fundamentally converge on inflammation. Peripheral inflammation significantly influences brain function and contributes to the development of neurological disorders, including Alzheimer’s disease, Parkinson’s disease, and multiple sclerosis. Understanding the role of inflammation in the pathogenesis of progressive neurological diseases is of crucial importance for developing effective treatments and interventions that can slow down or prevent disease progression, therefore, decreasing its social and economic burden.

## Introduction

Over the past 100 years, human life expectancy increased by 30 years, mainly due to scientific advances in several areas such as medicine and technology, and to the improvements in public healthcare and lifestyle (Olshansky 2018). However, in the past decades, the low death rates in combination with low birth rates have led to a shift in the distribution of countries’ populations toward older ages, especially in the Western world. This phenomenon, also known as the ‘aging population’, makes that the world’s population aged 60 and older will double by 2050 and reach 2.1 billion (Organization 2021). This growth in the elderly population brings unprecedented social and economic challenges and contributes to a steep rise in age-related diseases. The current knowledge shows that advanced age carries a higher risk for the development of diseases and disability, including cancer, cardiovascular disease, and neurologic disorders (Niccoli and Partridge 2012). Hence, a new clinical and scientific paradigm has emerged now focusing on healthy aging, to not just decrease morbidity but also increase the years of healthy living (Hansen-Kyle 2005). In a simplistic manner, biological aging results from the accumulation of broad-spectrum molecular and cellular damage over time as a result of the failure of cellular upkeep pathways (Niccoli and Partridge 2012; Partridge 2010). This leads to a gradual decline in physical and mental capacities. While aging is not a disease in itself, aging increases the predisposition for various diseases (Bruins et al. 2019).

Several interconnected theories have been proposed regarding the mechanisms of aging, of which *inflammaging*, a concept coined by Franceschi and his group (Franceschi et al. 2000), is prevalent at present times. *Inflammaging* refers to low-grade, prolonged, systemic inflammation throughout life/aging i.e., increasing pro-inflammatory status with advancing age (Kirkwood 2018). Immune system dysfunction occurs with age often referred to as “immunosenescence” (Lian et al. 2020). This process is characterized mainly by the decreased output of T cells (Lazuardi et al. 2005), decreased ability to attack infections or cancer, enhanced activation of the nuclear factor kappa B (NF-κB) transcription factor (López-Otín et al. 2013), extreme reactivity to new antigens, increased occurrence of autoimmune responses and the low-grade inflammation mentioned earlier and known as *inflammaging* (Hardeland et al. 2015; Santoro et al. 2018). The upregulation of the inflammatory response that accompanies aging results from the cumulative antigenic load throughout the lifetime but also from genetic predisposition, environmental exposure, and lifestyle (Deleidi et al. 2015), being of complex interpretation. One should keep in mind that inflammation is not only a main determinant of biological aging but also a shared outcome of other molecular pathways capable of originating inflammatory stimuli (Franceschi et al. 2017).

The incidence of neurological disorders has been growing considerably in the past decades. Globally, they represent the leading cause of disability and are ranked second as the leading cause of death after cardiovascular diseases (Feigin et al. 2020). Neurological disorders are a group of pathological conditions that involve both the central and peripheral nervous system. They are characterized by structural, neurochemical, and electrophysiological dysfunctions that culminate in the loss of neurons or neuronal networking (Farooqui 2016; Thakur et al. 2016). While the etiology of neurologic disorders differs and has complex multifactorial factors, in the end, inflammation and, especially, neuroinflammation seem to be the common denominator (DeMaio et al. 2022).

Inflammation not only makes the central nervous system (CNS) more susceptible to age-related damage but further contributes to exacerbating the cycle of age-related CNS decline. Aging and the devolvement of neurologic disorders in older adults share some basic pathways that fundamentally seem to converge on inflammation. Furthermore, while the contemporary lifestyle has allowed for an increased lifespan, it also brought many health issues due to increased exposure to pro-inflammatory sources (Gurusamy and Rajasingh 2019). The increase on chronic diseases is largely attributed to lifestyle changes including sedentary habits, processed food and drug consumption, elevated psychological stress, and exposure to environmental toxins and pesticides in food (Ganu et al. 2012). These environmental factors increase the vulnerability to chronic inflammatory diseases such as cardiovascular disease, type 2 diabetes, and some types of cancer. Despite a consistent upward trend in life expectancy estimates over the past two centuries, projections suggest that this trend may be reversed in future generations due to the growing prevalence of these chronic conditions (Anderson and Durstine 2019). Furthermore, environmental exposure to air pollution and pesticides, but also one’s lifestyle choices significantly increase the risk of developing neurological disorders (Chin-Chan et al. 2015). Inflammation is at the center of many age-related diseases and its involvement in neurologic disorders has been a centerpiece of many discussions that will be approached next.

Taking this into account, it is essential to understand how the chronic low-grade inflammation associated with aging contributes to the development of neurological disorders. After a brief explanation of immune and CNS dysfunctions featuring aging, this review will address in detail the inflammatory factors that possibly contribute to neuroinflammation and may culminate in neurological disorders. Considering the broad spectrum of neurological disorders and the impossible task to tackle all the diseases, this work will focus on the non-communicable progressive neurological disorders presenting the estimated highest rate of incidence, namely Alzheimer’s disease (AD), Parkinson’s disease (PD), and multiple sclerosis (MS) (Feigin et al. 2019).

## Inflammation and the central nervous system

For many years, the CNS was considered to be immune-privileged. However, a new understanding of both the immune system and CNS revealed that they share bi-directional communication, and the concept of immune privilege needs to be redefined. First, the CNS is immune competent due to the resident immunomodulatory glial cells that include microglia (tissue-resident macrophages), astrocytes, oligodendrocytes (Carson et al. 2006), and neurons (of note, although neurons are not always recognized as immune cells, they contribute to the neuroimmune response by interacting with immune effectors) (Tian et al. 2009). Contrary to what has been the past notion, T lymphocytes actually can migrate into the healthy CNS directly into the cerebrospinal fluid (CSF) via the choroid plexus (Strazielle et al. 2016) and inhabit the meningeal spaces providing immune surveillance (Filiano et al. 2017). Recently, it was also discovered a system of meningeal lymphatic drainage that clears cellular and insoluble components from the CSF and interstitial fluid (ISF) in the brain parenchyma to the deep cervical lymphatic nodes (Albayram et al. 2022). It is estimated that approximately 750,000 peripheral immune cells exist in the CSF, of which 90% are T cells and the remaining are B cells and monocytes. Therefore, peripheral immune cells are constantly in contact with CNS antigens and other factors (Agrawal et al. 2022).

In addition to the aforementioned pathways, both systems can also communicate through inflammatory mediators, namely cytokines. Cytokines are small peptides released in response to injury or infection, mostly by macrophages, monocytes, or nonimmune cells (fibroblasts and endothelial cells). They trigger several pathways both pro- and anti-inflammatory, to engage the immune challenge. Cytokines have a unique immunomodulatory profile due to their pleiotropy characteristics; in addition, cytokines can inhibit, induce, and control the action of other cytokines (Dias-Carvalho et al. 2022; Yarlagadda et al. 2009). Cytokines can be divided into three categories: pro-inflammatory or responsible for the upregulation of the immune response [e.g., interleukin (IL)-1, IL-6, and tumor necrosis factor alpha (TNF-α)]; anti-inflammatory or that damper the immune response (e.g., IL-4, IL-10, and IL-3) and hematopoietic that control the differentiation of hematopoietic cells [e.g., IL-3, IL-5, and granulocyte colony-stimulating factor (G-CSF)] (Yarlagadda et al. 2009). Most interestingly, peripheral pro-inflammatory cytokines can affect the CNS, either by easily crossing the blood–brain barrier (BBB) (Banks et al. 2009) or by entering through the brain’s more permeable circumventricular organs (Walker 2018). Moreover, the pro-inflammatory cytokines such as TNF-α, IL-6, and IL-1β can damage the BBB by destroying tight junctions of the neurovascular unit and, therefore, altering its permeability (Banks et al. 2009; Disdier et al. 2017). Once in the brain, these molecules can determine the resting microglia to change into an active state and produce pro-inflammatory mediators in a complex process called neuroinflammation. During neuroinflammation, microglia interacts with astrocytes via secretion of TNF-α, IL-1α, and complement 1q that prompts astrocytes to decrease phagocytosis and expression of neurotrophic factors (Koyama et al. 2022). Actually, astrocytes being the most abundant glial cell in the CNS are key players in the development of neuroinflammation (Matias et al. 2019). They also express transporters for glutamate, gamma-aminobutyric acid (GABA), and glycine that help to shape synaptic transmission (Osborn et al. 2016). Upon stimulation, astrocytes can undergo morphological and functional changes, increase homeostatic and trophic functions, increase proliferation and migration and increase their secretory activity (Buffo et al. 2010), resulting in increased expression of the glial fibrillary acidic protein (GFAP). This protein is usually used as a marker of this overall reactive process called astrogliosis (Matias et al. 2019). This reactive phenotype can be induced by increased levels of interferon-gamma (IFN-γ), IL-1β, IL-6, and TNF-α and, besides the morphological changes, it leads to the activation of the NF-κB pathway, the production of reactive oxygen species (ROS) and nitric oxide (^•^NO) and further secretion of IL-1β, IL-6, and TNF-α (Dá Mesquita et al. 2016). Reactive astrocytes have enlarged cell body sizes with thick branching of astrocytic processes. As mentioned, this is often evident by the increase in the filament protein GFAP and intracellular signaling calcium-binding protein B (S100B) (Simpson et al. 2010).

Simplistically, neuroinflammation is the activation of the CNS innate immune system in response to stressors, prompting the activation of glia, the release of inflammatory mediators, and the production of reactive oxygen /nitrogen species (ROS/RNS). It can be beneficial for the organism, resulting in tissue repair, enhanced neuroplasticity, and neuroprotection (DiSabato et al. 2016). However, most times, unresolved (neuro)inflammation can lead to deleterious consequences. The constant activation of glial cells (mainly astrocytes and microglia) in the inflammatory process diverts them from essential brain upkeep (Triviño and von Bernhardi 2021). Moreover, the ability of the brain to regenerate is limited. The brain is a highly organized structure that comprise neurons, mostly post-mitotic cells and, despite adult neurogenesis happening in the subventricular zone and the subgranular zone of the hippocampal dentate gyrus, these newly formed neurons are integrated only into memory-forming circuits. Thus, the brain is not well-equipped to cope with constant insults, and regeneration is limited (Valero et al. 2017). Furthermore, prolonged neuroinflammation alters the membrane expression of several neurotransmitter receptors, such as glutamate and GABA, which may result in impaired brain function, namely spatial learning, cognitive and motor dysfunction (Jacqueline et al. 2017).

Neuroinflammation leads to cellular impairment in various degrees, mainly synaptic dysfunction (Chugh et al. 2015), inhibition of neurogenesis (Connolly et al. 2022), microglial priming (Mumaw et al. 2016), apoptosis (Javadpour et al. 2021; Xu et al. 2007), abnormal phosphorylation (Soto-Faguás et al. 2021), cleavage (Bi et al. 2021), and aggregation (Lodeiro et al. 2017) of key functional CNS proteins. All these alterations result in accelerated brain aging and cognitive impairment (Jardanhazi-Kurutz et al. 2010). Elderly people are particularly vulnerable to neuroinflammation and its consequences, due to age-related alterations in the CNS, similar to what happens in the immune system and other age-related cellular changes (Chinta et al. 2015).

## Aging of the central nervous system

Even in older healthy individuals, there are several brain hallmarks of aging. The CNS barriers become more permeable with age due to morphological changes and loss of integrity of the neurovascular unit (Chu and Praticò 2009). That hyperpermeability results in higher infiltrations of circulating T cells into the white matter parenchyma (Batterman et al. 2021), which can contribute to neuroinflammation. Moreover, efflux transporters’ expression is decreased, making the removal of potentially neurotoxic substances deficient (Gorlé et al. 2016). As previously mentioned, the increase in circulating pro-inflammatory cytokines with advanced age, combined with their capacity to disrupt the neurovascular unit, may cause further damage (Disdier et al. 2017). Furthermore, higher levels of circulating pro-inflammatory cytokines were negatively correlated with brain volume in non-demented older adults (Zhang et al. 2016). The grey matter volume of the hippocampal formation also negatively correlates with circulating levels of IL-6 (Marsland et al. 2008), which could be associated with increased inflammatory state in aging. Moreover, advanced age is associated with continuous deterioration of white matter, evident by demyelination, axonal damage, and eventual neuronal loss, leading to disruption of white matter microstructural organization and the development of lesions (Raz et al. 2012). Regarding grey matter, its volume tends to decrease with age due to the decrease in volume and number of neurons (Sukhorukov et al. 2022). In addition, neuroglial cells suffer cellular senescence, which is characterized by loss of mitotic activity, changes in gene and epigenetic expression and in mitochondrial homeostasis, and also impaired redox state, and disruption of energetic metabolism (Maciel-Barón et al. 2018). These alterations significantly alter the function of the neuroglial cells, therefore, impacting the brain’s function. In particular, astrocytes take a special role in this by being responsible for homeostatic control, neuronal support, and regulation of synapses in coordination with microglia (Verkerke et al. 2021). In addition, astrocytes end-feet are integrated into the BBB and blood–cerebral spinal barrier (López-Teros et al. 2022). Although astrocyte senescence shares some common features with astrogliosis, the secretion of pro-inflammatory factors is smaller, being the senescent phenotype more consistent with chronically low levels of inflammatory markers (López-Teros et al. 2022; Verkerke et al. 2021). Considering the age-related alterations in microglia, several morphological changes have been observed, such as decreased arborization and shorter processes, abnormal cytoplasmatic structures; accompanied by functional deterioration, inflammatory hyperresponsiveness, and a tendency to form clumps, particularly in the white matter (von Bernhardi et al. 2010). Aged microglia also contain lysosomal inclusions of insoluble myelin fragments that contribute to microglial senescence and dysregulation (Colonna and Butovsky 2017). The co-existence of these two phenomena (increasing pro-inflammatory status and cellular senescence) in the brain contributes to the vicious cycle of inflammation that enhances brain damage with consequent cognitive decline.

## Alzheimer’s disease

The most prevalent type of dementia is AD, accounting for 60–80% of all dementia cases (Chu et al. 2022). In 2019, it was estimated to affect 50 million people worldwide and the number is projected to increase by 70% by 2050 (Nichols et al. 2022). Ninety percent of the cases affect older adults over the age of 65 years, and the occurrence doubles every 5 years with an exponential time-dependent increase in the risk of AD (Trevisan et al. 2019). The symptoms include loss of cognitive functions such as memory, language, and executive function, which translates into difficulties in carrying out everyday tasks. With disease progression, the patients are no longer self-sufficient (Zhu et al. 2021), which brings a huge social and economic burden to relatives. The hallmarks of AD include exacerbated accumulation of β-amyloid plaques, hyperphosphorylation of tau, neurofibrillary tangles (NFTs), neuropil threads with concomitant astrogliosis and microglial activation (Kadir et al. 2011; Serrano-Pozo et al. 2011).

**Fig. 1:**
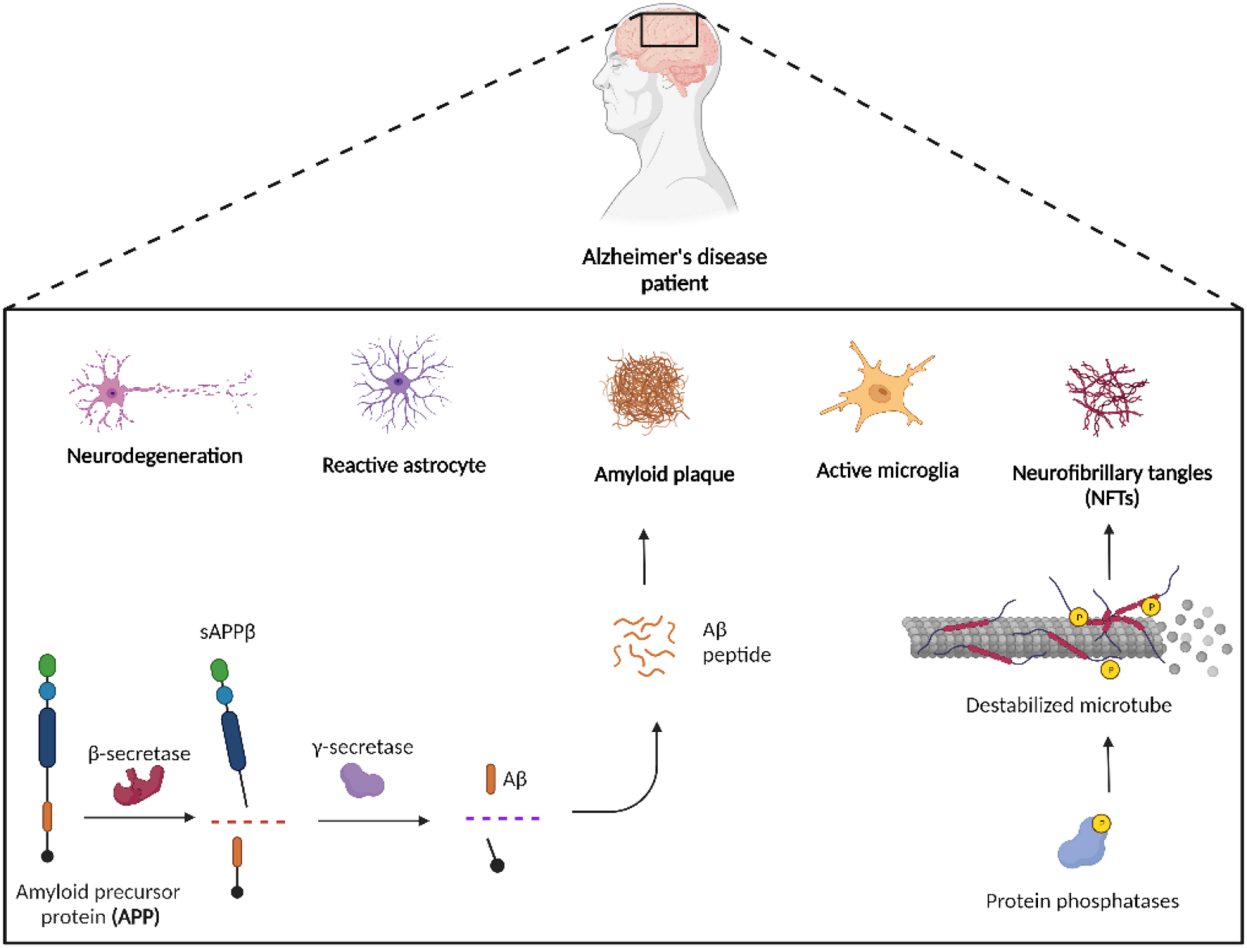
Neuropathological changes occurring in Alzheimer’s disease brain, emphasizing key features of the disease. Created with BioRender.com

Amyloid plaque build-up happens after differential processing of the amyloid precursor protein (APP). APP is a transmembrane protein expressed in many CNS cell types, harboring several cellular functions such as synaptogenesis and synaptic plasticity (Gralle and Ferreira 2007). This protein undergoes proteolytic modification via two alternative pathways: cleavage by α-secretase to generate the peptide sAPPα and a C83 carboxy-terminal fragment (non-amyloidogenic pathway) or cleavage by β-secretase to generate the peptide sAPPβ (amyloidogenic pathway). Following the β-secretase cleavage, further cleavage by γ-secretase releases extracellular peptides of different sizes, the most significant being the amyloid-β peptides (Aβ), usually ranging from 27 to 43 amino acids in length (Fig. 1) (Mukda et al. 2016). Interestingly, both sAPPβ and Aβ cause stimulation of axonal outgrowth (Chasseigneaux and Allinquant 2012). Neurons are the major source of Aβ; however, astrocytes also produce this molecule in smaller amounts, and since they outnumber neurons by five times, their contribution should not be discarded (Zhao et al. 2011).

While Aβ peptides, as sAPPα, have some essential neuronal functions, namely neurotrophic and neuroprotective effects, and stimulation of neural progenitor proliferation, excessive Aβ peptides are neurotoxic. To be clear, in a disease-free brain fully functioning, the rate of clearance does not allow its accumulation and aggregation (Lv et al. 2014). Nonetheless, the imbalance in the APP processing pathways that increase Aβ production and decrease the mechanisms of clearance, results in Aβ accumulation and consequential plaque formation (Yoon and Jo 2012). Pathogenic Aβ aggregation forms insoluble fibrils that cause neuronal dysfunction (Knopman et al. 2021). These aggregates are the “building blocks” of the prototypical AD senile plaques or neuritic plaques that comprise not only Aβ aggregates but also other proteins, such as apolipoprotein E (ApoE), elements of the complement cascade, and cytokines. Furthermore, senile plaques also include dystrophic neurites, reactive astroglia, and microglia (Knopman et al. 2021).

Regarding other pathognomonic signs, intracellular NFTs are composed of hyperphosphorylated forms of the microtubule-associated protein Tau (Fig. 1). This protein is present mostly in the axons of the neurons and is responsible for stabilizing cytoskeleton microtubules, having a key role in axonal transport and microtubule dynamics (Joseph et al. 2017). Multiple factors may contribute to the hyperphosphorylation of Tau such as extracellular amyloid plaques, dysregulation of phosphorylation, and compromised brain glucose metabolism. This leads to protein phosphatases activation, glycogen-synthase kinase-3β (GSK-3β), and cyclin-dependent protein kinase 5 (CDK5) being primarily responsible for the hyperphosphorylation of tau. However, other kinases such as protein kinase C (PKC), protein kinase A (PKA), and stress-activated protein kinases may also be involved (Gong and Iqbal 2008). The addition of a phosphate group to the Tau protein by activated kinases (Gong and Iqbal 2008) leads to Tau detachment from the microtubule with consequential microtubule instability and oligomerization of Tau, ultimately leading to the formation of NFTs (Ashrafian et al. 2021). The pathological association between Aβ and hyperphosphorylation of Tau is not fully understood yet. It appears that in AD, the dysregulation of Aβ precedes the Tau hyperphosphorylation (Blasko et al. 2004). All these abnormal protein misfolding triggers subsequent neuronal dysfunction and neuronal death, particularly in the hippocampus, entorhinal and temporoparietal cortices.

Despite “Aβ hypothesis” being widely accepted and self-explanatory to some of the underlying mechanisms of this disease, it does explain all of them. For example, Aβ deposition levels do not correlate with clinical manifestations, as senile plaques are also found in aged individuals without clinical manifestation of AD (Morishima-Kawashima et al. 2000). Furthermore, plaque formation does not always result in synaptotoxicity (Mucke et al. 2000). Therefore, other factors must trigger the cascade of protein malfunctioning. Genetic predisposition has been implicated in the risk of the development of AD. The apolipoprotein-e4 (*APOE-e4*) gene is the gene with the strongest impact on the risk of developing AD. This gene expresses the plasmatic ApoE protein involved in lipid and lipoprotein metabolism, but it also plays a role in stress response pending on the isoform involved. The allele ƐA is associated with an increased risk for AD (Dose et al. 2016). Although the mechanism is not fully understood, data suggest that the ƐA isoform causes altered lipid homeostasis resulting in increased unsaturation of fatty acids (Sienski et al. 2021). Moreover, the phenotype generated by *APOE-e4* leads to the breakdown of the BBB by activating a pro-inflammatory cascade involving the increase of the inflammatory protein cyclophilin A. The weakening of the BBB after this inflammatory cascade allows the passage and neuronal uptake of many blood-circulating neurotoxins and reduces microvascular and cerebral blood flow (Bell et al. 2012).

## Inflammation and Alzheimer’s disease

Generally, Aβ deposition and NFTs are regarded as the first events responsible for cognitive decline and brain atrophy in AD patients. Both protein aggregates are potent activators of the immune system (Blasko et al. 2004). Another hint of immune involvement in AD was revealed by a study showing that the AD patients’ cortex had a higher percentage of reactive microglia compared to age-matched controls (Carpenter et al. 1993). Higher levels of the pro-inflammatory IL-1β were also found in the frontal, parietal and temporal cortices, hypothalamus, thalamus, and hippocampal formation of AD post-mortem samples when compared to age-matched control individuals (Cacabelos et al. 1994). Brain inflammation is widely observed in AD patients as shown by microglial activation and astrogliosis. This phenomenon has been considered secondary to neurodegeneration but further research on the causal order in AD has revealed abnormal inflammatory signaling in the brain, including glial activation in pre-symptomatic AD (Chun and Lee 2018). In addition, an in vivo study demonstrated that prenatal exposure to a viral cytokine inducer led to increased deposition of APP and its proteolytic fragments, Tau aggregation, microglia activation, and reactive gliosis (Krstic et al. 2012). In 1990, Bauer and colleagues showed that in cell-cultured neurons alpha-2 macroglobulin (α-2 M), a proteolytic protein, interferes with the normal cleavage of APP, thus promoting the cleavage that originates Aβ peptides. However, the striking finding was that the pro-inflammatory IL-6 stimulated the synthesis of α-2 M, contributing to a higher yield of Aβ (Bauer et al. 1991). The authors also analyzed post-mortem samples of isocortical and hippocampal areas of AD patients and found that senile plaques displayed immunoreactivity for IL-6 and α-2 M, while no immunoreactivity to these proteins was found in the brain of age-matched controls. The authors suggested that AD might start with a sequence of immunological events beginning with increased levels of IL-6 that stimulates the production of α-2 M, resulting in APP-altered cleavage (Bauer et al. 1991).

The levels of blood and serum pro-inflammatory cytokines have been investigated in AD patients, but the obtained results have been contradictory. Nevertheless, meta-analysis and systematic review of 170 studies, where peripheral inflammatory markers were assessed, revealed significantly altered levels of inflammatory markers in AD patients (Shen et al. 2019). Serum pro-inflammatory (IL-1β and IL-2) cytokines are elevated mainly in early-stage (AD with mild cognitive impairment), in comparison to healthy controls and more advanced AD cases (King et al. 2018). Furthermore, isolated human lymphocytes stimulated with Aβ (concentration ranging from 10^–8^ to 10^–5^ mol/L) secrete cytokines in a biphasic manner: in the first 24 h of exposure, there is a marked increase in all assessed cytokines for all concentrations of Aβ, possibly to help lymphocytes to eliminate the threat, that is followed by a slight decline (Teixeira et al. 2008). It is reasonable to speculate that peripheral pro-inflammatory mediators are implicated in the initial steps of AD and may contribute to the progression of the disease; however, other regulatory aspects may be involved.

It is a common knowledge that peripheral circulating cytokines can interfere with brain processes and stimulate local immune effectors and, in the AD brain, extensive active microglia and reactive astrocytes have been commonly observed (Sakakibara et al. 2019). As previously mentioned, microglia are CNS resident immune cells and can modulate a broad spectrum of cellular responses. During injury, microglia swiftly extend processes and then migrate to the lesion site, identify the immune stimulus/stimuli, ramify the processes, and set in place an immune response to eliminate the threat (Ransohoff and Perry 2009). Regarding AD, microglia can phagocyte Aβ readily, but whether internalized Aβ can be degraded or result in other processes is still unclear. A study found that microglia retain Aβ for long periods without degrading the peptides in comparison with two other proteins (acetylated low-density lipoprotein and α-2 M), suggesting that microglia can incorporate large amounts of Aβ and the indigested material causes engorged cell bodies (Paresce et al. 1997). A later study demonstrated that nonactivated microglia were unable to degrade Aβ, but microglia stimulated with macrophage colony-stimulating factor (M-CSF) were cable of degradation of Aβ through lysosomes (Majumdar et al. 2007). Despite this, the inflammatory cascade that both Aβ and activated microglia cause, outpaces the effort to eliminate Aβ.

Astrocytes significantly outnumber microglia (Blasko et al. 2004), and it is important to highlight their importance in the development and progression of AD. High levels of GFAP and S100B have been found in the CSF of AD patients (Fukuyama et al. 2001; Petzold et al. 2003), highlighting the role of reactive astrocytes in AD progression. In contrast with microglia, astrocytes can degrade Aβ without mediators. Studies in mice overexpressing Aβ showed that astrocytes produce small amounts of the Aβ-degrading enzyme neprilysin and this production decreases with age (Apelt et al. 2003). Neprilysin is an important neuropeptidase, which can modulate brain function by controlling the metabolism of sensory and inflammatory neuropeptides including tachykinins and neurokinins (Nalivaeva et al. 2020). Furthermore, this enzyme is the primary enzyme responsible for breaking down Aβ peptides. In the transgenic mice model of AD overexpressing neprilysin, a reduction of Aβ production, reduction of inflammatory biomarkers, and improvements in cognition were detected (El-Amouri et al. 2008).

**Fig. 2:**
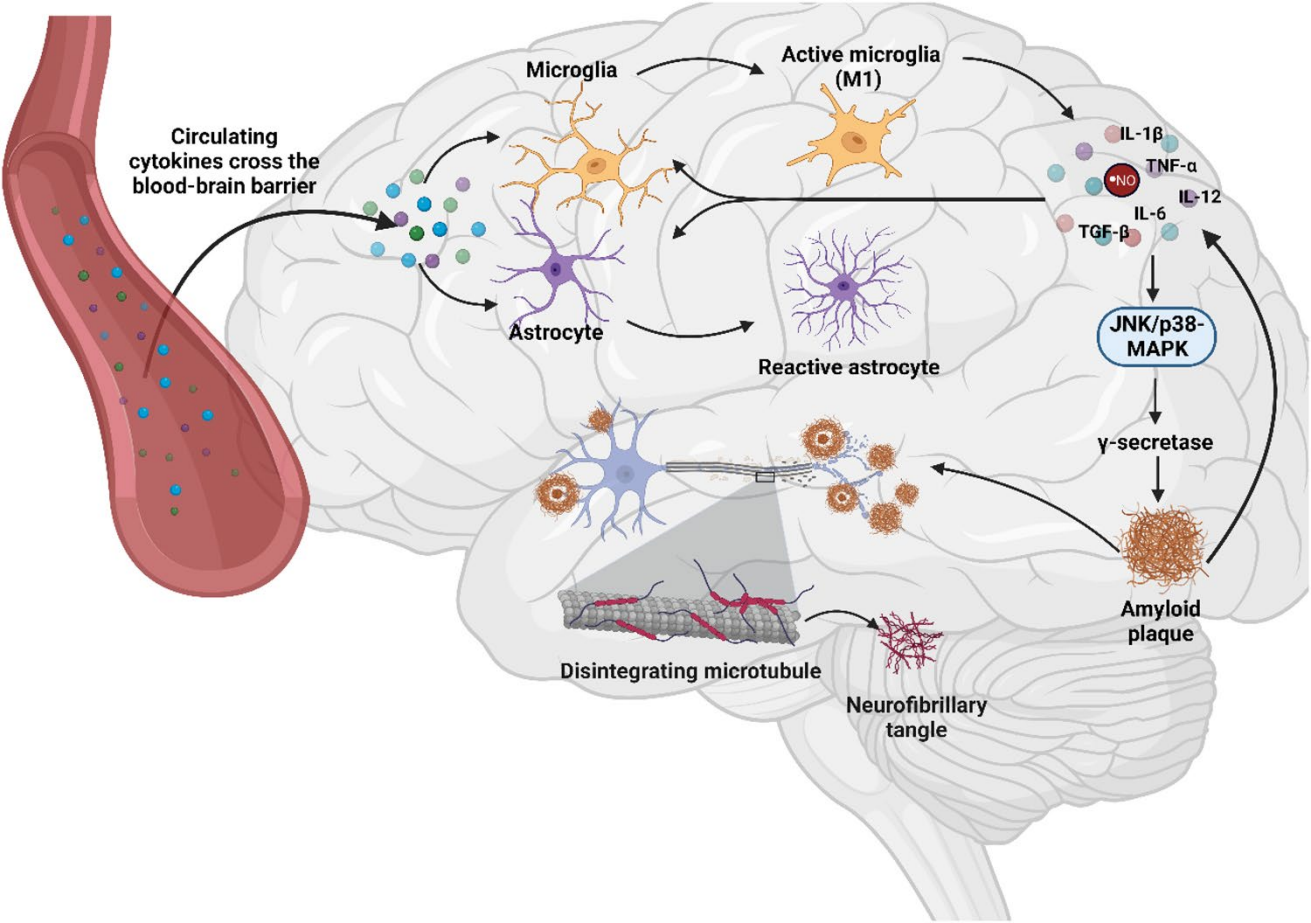
Schematic illustration of the sequence of events in Alzheimer’s disease (AD) and peripheral inflammation, emphasizing the interplay between these two processes. Circulating cytokines can cross the blood–brain barrier (BBB) and stimulate neuroglial cells (mainly astrocytes and microglia) to produce in situ more inflammatory mediators. Those inflammatory mediators can, in turn, stimulate other neuroglial cells, but also activate other signaling cascades such as the Jun N-terminal kinase (JNK) and p38 mitogen-activated protein kinase (MAPK). The JNK/p38-MAPK induces the activity of γ-secretase increasing the production of Aβ peptides and amyloid plaques. These plaques cause neurodegeneration and its accumulation, in the pro-inflammatory environment, leads to hyperphosphorylation of Tau and formation of the neurofibrillary tangles (NFTs). Created with BioRender.com

Not only can peripheral cytokines directly trigger immune effectors in the brain, but also some cytokines such as TNF-α, IL-1β, IFN-γ, IL-6, and transforming growth factor beta (TGF-β) can induce γ-secretase enzymatic activity through Jun N-terminal kinase (JNK) and p38 mitogen-activated protein kinase (MAPK) pathways, which cleaves APP and initiates Aβ formation (Fig. 2) (Liao et al. 2004). In turn, Aβ can further activate glial cells to produce more cytokines that can act on other CNS cells or as autocrine signals (González-Reyes et al. 2017), propagating the cycle of neuroinflammation and neurodegeneration that characterizes AD (Fig. 2).

## Inflammatory biomarkers

Early diagnosis or prognostics in AD is essential for disease management and even for valid attempts to try to delay the onset of symptoms. There has been an extensive search for appropriate biomarkers, easily accessible and specific to AD that could signal, with accuracy, the initial stages of AD (where therapies are more likely to have positive effects). Currently, CSF levels of Aβ peptides and hyperphosphorylated Tau are the commonly used biomarkers for AD (Molinuevo et al. 2013). However, the lumbar puncture required for CSF extraction is invasive. The markers are elevated when the pathophysiology of AD is fully set and the likelihood of preventing or improving neurodegeneration is small to none at that time (Morgan et al. 2019). Furthermore, Aβ is not AD specific since it is also produced in peripheral tissues including the pancreas, adipose tissues, skeletal muscles, and liver (Shigemori et al. 2022). Moreover, GFAP and neurofilament light chain are biomarkers of neuroinflammation and neuronal injury, respectively, but they are very unspecific and reported to be increased in other neurodegenerative diseases (Verberk et al. 2021).

As this work is set on the premise that inflammation is crucial for the initiation of AD, a larger focus on finding inflammatory biomarkers that could be linked to the development of AD is considered. In a cohort of elderly people (259 controls, 199 individuals with mild cognitive impairment, and 262 AD patients), the plasma levels of 53 inflammatory proteins were measured. The authors observed that 10 proteins were significantly different between groups. Between AD patients and controls, the levels of soluble complement receptor 1 (sCR1), two complement regulators (factor B and factor H), chemokines eotaxin-1, and monocyte chemoattractant protein-1 were significantly different (Morgan et al. 2019). Another study set the blood levels of inflammatory markers of 191 patients, for 7 years, where in the beginning the subjects were cognitively healthy but later developed AD. The results revealed that higher levels of tumor necrosis factor-alpha receptor-1 (TNFR1) were associated with a greater risk of mild cognitive impairment, associated with early-phase AD (Gross et al. 2019).

While there are some strong candidates, to date, there are no specific inflammatory biomarkers to accurately identify early stages of AD, nor strong inflammatory predictors. Rather, an overall increase in inflammatory status is considered a susceptibility factor for AD. Hitherto, the most reliable predictor of AD is still the genetic susceptibility of APOE-e4, being this a modulator of the immune response. In the brain, the inflammatory processes mediated by microglia and astrocytes are modified depending on the isoform of ApoE, with the isoform APOE-e4 producing the strongest pro-inflammatory reaction (Newcombe et al. 2018).

## Inflammation as target for Alzheimer’s disease

Targeting inflammation has yielded controversial results in AD treatment. While the use of non-steroidal anti-inflammatory drugs was associated with a lower risk of developing AD (Zhang et al. 2018), clinical trials aimed to access its effectiveness in AD patients failed to show any clinical benefit (Neurology 2007; Szekely et al. 2008). These results further support the concept that inflammation may be the initial trigger but once the inflammatory cascade starts, it is difficult to stop it. Even so, some data show that when it is tackled beforehand, there is less risk to develop AD in susceptible patients.

Currently, there are only five drugs approved for AD by the Food and Drug Administration (FDA), which only offer symptom management. These drugs are rivastigmine, galantamine, tacrine, and donepezil which are cholinesterase inhibitors, and memantine, an antagonist of the N-Methyl-D-Aspartate-receptor (Ali et al. 2019). In general, the drugs aim to increase acetylcholine, leading to increase neuronal communication (Birks 2006) and blocking glutamate, mainly released from active astrocytes. The inhibition of glutamate binding to the receptor prevents excessive activation that causes apoptosis (Wang and Reddy 2017). Nevertheless, these therapies are only symptomatic and have limited clinical efficacy. Recently, therapeutic approaches have focused on monoclonal antibodies targeting Aβ peptides. Aducanumab was approved by the FDA in 2021 for AD patients with mild cognitive impairment or mild dementia and demonstrated efficacy in decreasing Aβ plaques (Padda and Parmar 2022). Lecanemab (Leqembi) was granted accelerated approval in early 2023 by the FDA. This novel treatment reduced amyloid plaques in early AD patients and resulted in moderately less cognitive decline in a double-blind, phase 3 clinical trial (van Dyck et al. 2022). Both therapies have promising clinical trial results, but trials in the real world and large scenarios are needed to access their efficacy, only possible when are undertaken broader pharmacovigilance studies. Even if they prove some efficacy and related safety, the need to promote early diagnosis is key to allowing any of these therapies to have any chance of success.

## Parkinson’s disease

**Fig. 3:**
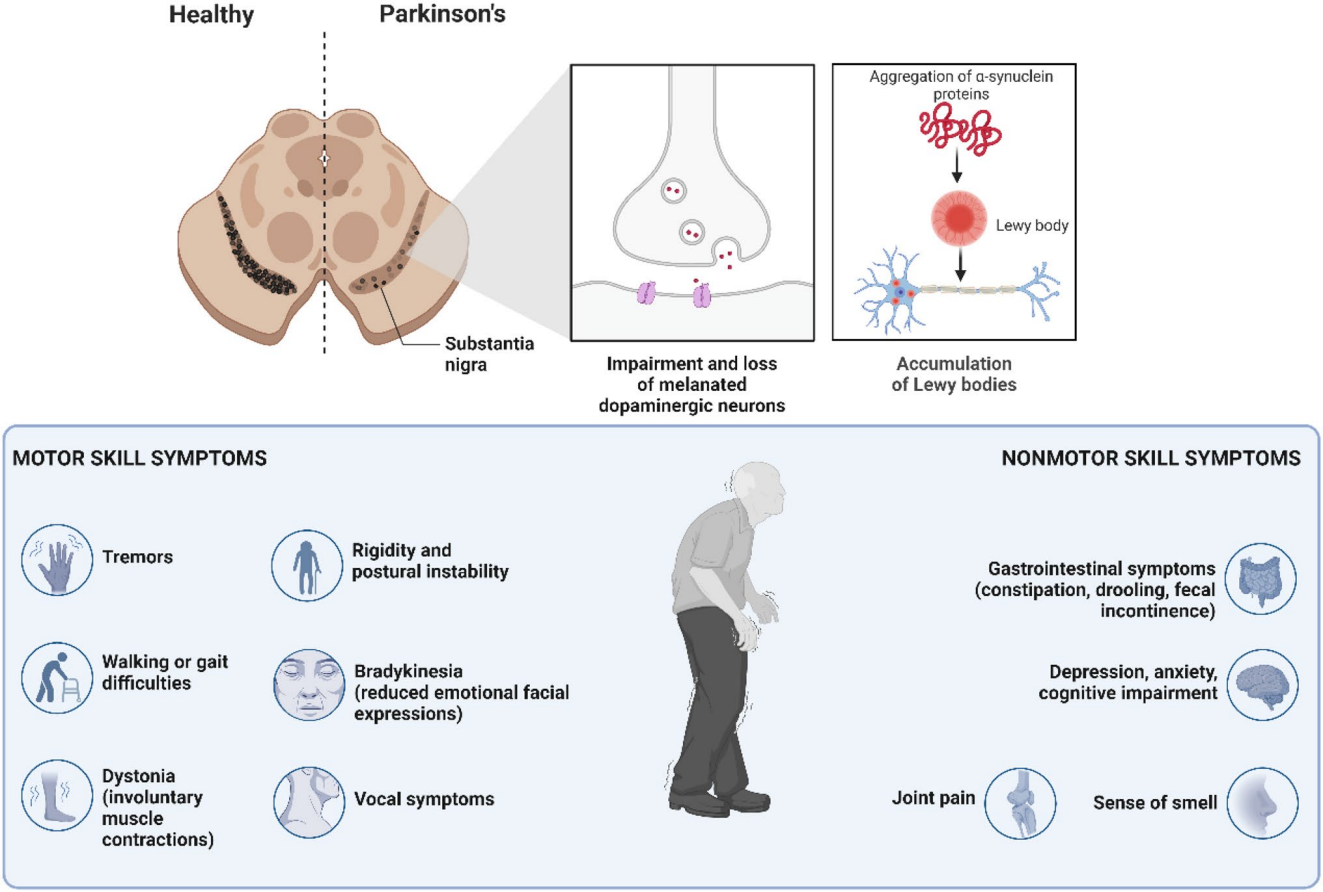
Illustrative representation of the effects of Parkinson’s disease (PD) in the brain and listing of motor and non-motor skill symptoms. Degeneration of pigmented dopaminergic neurons in the midbrain and misfolded α-synuclein and consequential accumulation of Lewis bodies are the hallmarks of PD. The neuronal loss causes dysfunction of the nigrostriatal pathway with a decrease in the levels of dopamine. Usually, this later pathway impairment results in the cardinal motor symptoms of PD. Created with BioRender.com

One to two individuals per 1000 of all ages are affected by PD; however, as with AD, the incidence risk increases with age, affecting 1% of the population above 60 years (Tysnes and Storstein 2017). It ranks as the second most common progressive neurologic disorder, being that heritable forms of PD account for only 5–10% of the cases (Poewe et al. 2017). In the past, this disease was considered solely as a movement disorder due to pathological tremors. PD physical symptoms include resting tremors, dyskinesia, ankylosis, and postural instability. Non-motor symptomatology can include cognitive impairment, dementia, depression, and sleep disorders, and, contrary to what was initially proposed, these symptoms can appear earlier than the physical ones (Zhu et al. 2022). PD is characterized by both loss of pigmented dopaminergic neurons in the *substantia nigra pars compacta* and deposition of α-synuclein in the cytoplasm of several types of neurons (Sian-Hulsmann and Riederer 2021). The degeneration of dopaminergic neurons in the *substantia nigra pars compacta* results in dopamine loss in the basal ganglia, an area responsible for coordinating fine motor skills (Wong and Krainc 2017). In the early stages of PD, the loss of dopaminergic neurons happens in the ventrolateral part of *substantia nigra*, and with time, the loss spreads to other dopaminergic neurons of the midbrain (Poewe et al. 2017). These neuropathological hallmarks are present in the idiopathic form of PD and rare inherited forms (Fig. 3).

α-Synuclein is a presynaptic protein, located in the proximity of synaptic vesicles. α-Synuclein is believed to play an important role in maintaining an adequate supply of synaptic vesicles and modulating synaptic transmission (Stefanis 2012). Intracellular homeostasis of this protein is maintained through the ubiquitin–proteasome and the lysosomal autophagic systems (McKinnon et al. 2020). Under certain circumstances, α-synuclein can natively unfold and generate β-sheet structures (similar to Aβ in AD). The initial “rings” of oligomers of α-synuclein, also known as protofibrils, become insoluble and start to merge into fibrils. Such fibrils are the main component of the cytosolic clumps termed “Lewy bodies” (Poewe et al. 2017; Stefanis 2012). The accumulation and aggregation of the fibrils can be due to overproduction of the protein, a decrease in their proteolysis and clearance, the presence of mutated forms of the protein that are more likely to aggregate, or impaired pathways of handling misfolded proteins (Stefanis 2012). Aging has been linked to lower proteolytic abilities, which, as stated, might contribute to the accumulation of damaged α-synuclein (Poewe et al. 2017). Compelling data exist suggesting that modified α-synuclein has a central role in the pathogenesis of PD. Those protein aggregates are highly neurotoxic. A study in transgenic mice expressing human α-synuclein showed that the overexpression of α-synuclein and formation of oligomers alone are enough to deteriorate the locus coeruleus neurons (located in the pons of the brainstem), cause fiber degradation and trigger behavioral deficits (Butkovich et al. 2020). While normal α-synuclein is located near the presynaptic vesicles, the misfolded oligomers and bigger clumps are located throughout the neuronal cytoplasm and neurites, which can hinder cellular function further than the presynaptic portion (Wong and Krainc 2017). These protein changes have been pointed as candidates to trigger the action of the immune system in PD as a response mechanism, while some authors believe that α-synuclein aggregation is, in fact, a consequence of the pro-inflammatory status developed with age and its compromised protein folding machinery (Galiano-Landeira et al. 2020).

## Inflammation and Parkinson’s disease

The etiology of non-hereditary PD is largely unknown. Considering the inherited forms, only 10% of the patients report positive family predisposition (Klein and Westenberger 2012). Two types of inherited genetic mutations can cause PD: autosomal dominant mutations (including *SNCA*, *LRRK2*, *VPS35*, and *EIF4G1*) and autosomal recessive mutations (such as *PARK2*, *PINK1*, and *DJ-1*) (Klein and Westenberger 2012; Stefanis 2012). Besides α-synuclein inclusions, an increasing bulk of the literature has pointed to the role of inflammation in PD as crucial. One of the first indications that inflammation may trigger PD was seen when patients infected with the viral pathogen influenza virus (encephalitis lethargica) displayed PD-similar symptoms (Lutters et al. 2018). Ever since that discovery, several patients with viral pathogenic infections were more thoroughly observed and PD symptomatology has been detected, suggesting the possibility that pathogens might reach the basal glia and trigger neuroinflammation leading to the development of PD (Pajares et al. 2020). On the other hand, intake of certain non-steroidal anti-inflammatory drugs in mid-life was linked with decreased risk for PD development (Wahner et al. 2007). Further evidence of the role of inflammation on PD was found after post-mortem analysis of the *substantia nigra* of PD patients, where a higher density of active microglia in comparison to age-matched healthy controls was found (Mirza et al. 2000). The *substantia nigra* presents a higher density of microglia among different brain regions, therefore, being more susceptible to inflammatory events and microglial activation. These aspects might cause damage to its’ dopaminergic neurons (Yan et al. 2014). Single nucleus RNA sequencing (snRNA-seq) analysis of human post-mortem midbrain tissue of PD patients also revealed disease-specific astrogliosis, microgliosis, and loss of oligodendrocytes in that area. While in healthy age-matched subjects, the astrocytes overexpressed CD44 (a marker for astrocyte-restricted precursor cells), the astrocytes of PD patients revealed a pro-inflammatory trail characterized by elevated gene expression of *IL1B*, *GPNMB*, and *HSP90AA1* (Smajić et al. 2022). Moreover, in those patients, astrogliosis was linked with upregulated genes of several heat-shock proteins that usually co-localize with α-synuclein accumulation (Smajić et al. 2022). To further understand the involvement of the immune system in PD, T cell infiltration in the *substantia nigra pars compacta* of human post-mortem brain tissue was evaluated on patients featuring Lewy Bodies disease (only α-synuclein clumps deposition, which is considered to be the pre-symptomatic stages of PD), in patients with established PD and age-matched healthy controls (Galiano-Landeira et al. 2020). In PD patients, the density of CD8-positive cells increased by 2.5-fold in comparison to healthy controls, indicating *substantia nigra pars compacta* T cell infiltration (Galiano-Landeira et al. 2020). Next, the authors investigated how these signs correlated with neuronal loss in PD. In the *substance nigra pars compacta* of PD patients, several cytotoxic T lymphocytes (CTL) surrounding or in contact with degenerating dopaminergic neurons were visible, being important to add that dopaminergic neurons can be targeted by CTL since they express major histocompatibility complex (MHC) class I (Cebrián et al. 2014). In addition, the higher density of CTL was positively correlated with the decreased density of dopaminergic neurons in PD patients (whose loss was more striking when compared with both the control and Lewis bodies group). The most striking finding was that patients with Lewis bodies disease with α-synuclein aggregation only in the olfactory bulb presented a higher density of CD8-positive cells than patients with Lewis bodies patients with α-synuclein clumps in the *substantia nigra pars compacta* and even patients with PD. These data suggest that CD8-positive cell infiltration precedes both α-synuclein aggregation and neuronal cell death (Galiano-Landeira et al. 2020). A study in a mouse model that carries the human A30P/A53T double-mutated α-synuclein gene, corroborated the correlation between increased T cells and loss of dopamine in the striatum. Furthermore, in the *substantia nigra*, the increase in CD8-positive T cells and the density of microglia was age-dependent (Rauschenberger et al. 2022).

As previously mentioned, PD has been associated with cases of viral infection. Braak and colleagues (Braak et al. 2003) hypothesized that sporadic PD is triggered by pathogens that enter the body via the nasal cavity and are lodged in the gut, meaning that PD can be triggered in either the neurons of the nasal cavity or in the neurons in the gut (Braak et al. 2003). They advocate that the microbial products disrupt the immune response, causing immune activation and misfolding of α-synuclein in the periphery. From there, the misfolded α-synuclein aggregates and spreads toward the CNS via the olfactory tract or the vagal nerve (Rietdijk et al. 2017). Clinical data have found Lewy bodies in the olfactory bulb, dorsal motor vagal nerve, and enteric nervous system (Sharma et al. 2019), reinforcing the possibility that disease spread from the periphery to the CNS and that, peripheral immune cells and not brain immune cells, might be the first ones to react to misfolded α-synuclein occurring in the periphery.

Most interesting, a usual toxin aiming to promote PD was used in different aged animals. In mice treated with a dopaminergic neurotoxin [1-methyl-4-phenyl-1,2,3,6-tetrahydropyridine (MPTP)], aged mice had higher loss of dopaminergic cells in the striatum compared to younger mice. In addition, the dopaminergic loss was correlated with inflammation and that area had increased levels of IL-6, IL-1β, TNF-α, IFN-γ, and TGF-β1 (Ciesielska et al. 2007). Of note, the older control mice had increased basal levels of IL-6 in comparison to younger mice, reinforcing the notion that the pro-inflammatory status is enhanced with age (Ciesielska et al. 2007), or at least favored by it. Resorting to the PD mice model obtained while using the selective dopaminergic neurotoxin 6-hydroxydopamine (6-OHDA), the authors found that peripheral administration of the inhibitor for soluble TNF-α significantly reduced neuronal dopaminergic loss in the *substantia nigra pars compacta* but also reduced microglia and astrocyte activation (Barnum et al. 2014).

Regarding the pathways linked to inflammation, the Janus kinase (JAK)-signal transducer and activator of transcription (STAT) pathway is triggered upon cytokines, ILs, and growth factors action on their respective family of transmembrane receptors (Seif et al. 2017). This pathway is vital in regulating the development, differentiation, and function of adaptive and innate immune cells. Its dysregulation, particularly by activating and depolarizing myeloid cells and T cells, has been linked with several inflammatory disorders (Qin et al. 2016). In primary macrophage and microglial cultures, exposure to α-synuclein results in the activation of the JAK/STAT pathway and induced the expression of inducible ^•^NO synthase (iNOS), IL-6, TNF-α, and MHC class II (Qin et al. 2016). On the other hand, in rats overexpressing α-synuclein, pharmacological inhibition of the JAK/STAT pathway suppresses microglia activation, decreases expression of pro-inflammatory markers, and reduces infiltration of T cells in the *substantia nigra* (Qin et al. 2016).

NF-κB is also involved in regulating aspects of the immune system and inflammatory responses. This transcription factor responds to many immune mediators and once activated, the cascade of events can lead to the upregulation of various pro-inflammatory genes (Singh et al. 2020). Ghosh and colleagues (Ghosh et al. 2007) found NF-κB activation in the *substantia nigra pars compacta* of both the MPTP-mice model of PD and in PD patients. Furthermore, in the PD mice model, pharmacological inactivation of this inflammatory transcription factor pathway led to a decrease in dopaminergic neuronal loss, improved locomotor functions, and decreased expression of several pro-inflammatory molecules in the midbrain (Ghosh et al. 2007).

From the first evidence of inflammatory involvement in PD to the current theories, there has been a shift in the point of view. Most of the first studies on PD were focused on the degeneration of the nigrostriatal pathway, where neuronal death was considered the trigger for inflammation. However, a more holistic approach to PD allowed for a better understanding of how the immune system responds in association with PD. Now, there is evidence that inflammation, in particular, peripheral inflammation could be the starting point for PD leading to neuroinflammation. It was shown that PD initiation might occur at an early stage in life, with the involvement of both peripheral and brain immune cells. In fact, the neuronal dysfunction is not limited to the *substantia nigra* and α-synuclein may be a “byproduct” of the inflammatory environment.

## Inflammatory biomarkers

Classical clinical biomarkers for PD include subtle motor dysfunction. Some of these pre-PD symptoms include rapid eye movement sleep behavior disorder, hyposmia (partial or complete loss of the sense of smell), constipation, and mood disorders (Li and Le 2020). While rapid eye movement sleep behavior disorder is strongly associated with the risk of developing PD (Iranzo et al. 2013), in most cases, it requires self-awareness of sleep disorders by the individual and proper identification of the disorder by a clinician. Imaging biomarkers, mainly dopamine transporter single-photon emission computed tomography and fluorodopa positron emission tomography, have been used to detect changes in the dopaminergic system with high sensitivity and specificity (Suwijn et al. 2015). Nevertheless, these methods are not widely available and are only useful when the dopaminergic system is already failing, and not as much in an earlier stage PD. As stated, α-synuclein is a major component of PD, and CSF levels of α-synuclein have been widely accepted as a diagnostic biomarker of PD (Hong et al. 2010). Once again, this biomarker presents several limitations due to the difficulty to obtain CSF, being a highly invasive procedure. On the other hand, the blood concentration of α-synuclein varies greatly between PD patients, and no correlation has been established yet (Li and Le 2020).

Clinical research has been nowadays focused on identifying a prodrome inflammatory biomarker for PD. While in the past, many possible peripheral biomarkers have emerged, their accuracy, sensitivity, and specificity need to be accessed in larger cohorts of patients to prove their routine clinical value. In a longitudinal study, a positive correlation between increased blood levels of inflammatory chemokines CXCL12 and CX3CL1 and pro-inflammatory cytokine IL-8 and increased risk of PD was found (Li et al. 2022). Xu and colleagues measured serum levels of several inflammatory markers and found that PD patients had considerably higher levels of inflammatory cytokines IL-1β and IL-33 in comparison to healthy controls. In the early-stage PD subgroup, the levels of these cytokines were even higher than in the late-stage PD subgroup (Xu et al. 2022). Moreover, serum levels of soluble TNFR1 were also increased in a sample of PD patients in comparison to healthy controls (Scalzo et al. 2009). Nevertheless, elevated pro-inflammatory cytokines are a broad indication for several diseases. Thus, researchers have focused on finding a particular cytokine profile in combination with other biomarkers. Li and colleagues measured the nuclear receptor related 1 protein (NURR1), a transcription factor important for maintaining dopaminergic neurons and regulating neuroinflammation, and cytokines levels in the peripheral blood mononuclear cells in a cohort of 312 PD patients, 318 healthy control and 332 non-PD neurological disease control (Li et al. 2018). The authors observed that NURR1 levels were lower in PD patients in comparison with the other groups. Furthermore, TNF-α, IL-1β, IL-6, and IL-10 levels were significantly higher in PD in comparison to both control groups, and their levels were negatively correlated with the levels of NURR1. These results indicate that lower levels of NURR1 in combination with higher levels of pro-inflammatory cytokines could suggest PD presence and the NURR1 should be investigated as a possible predictor of PD (Li et al. 2018). Eidson and colleagues also sought to find a serum panel of inflammatory markers to monitor inflammation and disease progression. They observed that serum IFN-γ, TNF-α, and neutrophil gelatinase-associated lipocalin (NGAL) were significantly different and stable across 24 h when comparing PD patients and healthy controls (Eidson et al. 2017).

## Inflammation as a target for Parkinson’s disease

Regarding the pharmacological treatment of PD, its management consists mostly of drugs linked to dopamine pathways (Poewe et al. 2017). The most widely used is l-3,4-dihydroxyphenylalanine (L-DOPA), which is the precursor for dopamine synthesis. Catechol-O-methyltransferase inhibitors are also used in combination to prevent dopamine’s peripheral metabolism and, therefore, increase L-DOPA bioavailability (Armstrong and Okun 2020). Glial cells can also clear dopamine in the synaptic cleft via monoamine oxidase type B, so inhibitors for this enzyme prolong and increase synaptic dopamine levels by hampering its metabolization (Poewe et al. 2017). As last resort therapies, dopamine agonists can also be a therapeutic choice to help decrease motor symptoms by providing continuous dopaminergic stimulation, even when dopamine production is largely impaired (Antonini et al. 2009).

In the past years, some disease-modified therapies that target α-synuclein and its pathway have been investigated, as well as susceptible genes and proteins implicated in PD. The research has focused on vaccines, antibody therapy, and immune-mediated therapies to clear abnormal protein aggregates. There are several ongoing clinical trials and, in 2019, FDA approved the drug istradefylline (Nourianz) (Administration USFaD 2019), a selective adenosine A2a receptor antagonist that inhibits T CD4 cell hypersecretion of IL-17A and IL-8 (Tokano et al. 2022). Considering other therapies targeting inflammation, as previously mentioned, non-steroidal anti-inflammatory drugs were correlated with reduced risk of developing PD (Wahner et al. 2007), while there are no grand-scale clinical trials to confirm this association. Nevertheless, sargramostim, a recombinant form of human granulocyte–macrophage colony-stimulating factor (GM-CSF) FDA-approved for cancer patients, has been used in PD patients as an anti-inflammatory therapy, and so far, it has yielded great results, mainly improved motor and cortical activities (Gendelman et al. 2017). Furthermore, inhibition of the enzyme myeloperoxidase, which is involved in inflammation and degeneration, in microglial cells, by the selective and irreversible inhibitor AZD3241, was able to repress microglia and protect dopaminergic neurons and proved to be safe and well-tolerated in phases I and II of clinical trials (Jucaite et al. 2015); however, more studies are required specially on inflammatory pathways that can characterize PD. Overall, these data indicate that α-synuclein is linked to inflammatory cascades, and targeting them might slow down the progression of PD.

## Multiple sclerosis

A chronic inflammatory and neurodegenerative disorder of the CNS, MS is characterized by its’ autoimmune nature (Temmerman et al. 2022). This means that the immune system erroneously targets and attacks the myelin sheath in the brain and spinal cord, leading to demyelination of the CNS. In 2020, MS was estimated to affect 2.8 million people worldwide, which means that 1 in every 3000 people in the world is living with MS (Gbaguidi et al. 2022). Clinical manifestations of MS are non-specific and include visual loss, loss of strength/power in an arm or leg, a sense of numbness in the legs, fatigue, and cognitive impairment (Stoiloudis et al. 2022). To date, there is no clear cause for the development of MS but it seems to result from an interplay of environmental exposures, lifestyle, and genetics, and not a single causal event. Some of the known risk factors to consider to develop MS are sex, geographical location (usually higher latitudes are associated with more cases of MS), previous infection with the Epstein–Barr virus, smoking, a higher percentage of body fat, and lack of vitamin D (Ramagopalan et al. 2010). MS has a higher incidence in women than in men (approximately 3 to 1), suggesting that sex hormones might have some influence (Voskuhl et al. 2020). Regarding genetics, MS is not hereditary, but genome-wide studies showed that more than 230 risk alleles have been identified, mostly related to the immune system, that may impact MS pathology. The human leukocyte antigen (HLA) variations, which encode cell-surface MHC proteins, exert a strong effect on MS, but how that unfolds is still not clear (Alcina et al. 2012).

The starting event that leads to pathologic symptoms of MS seems to be the infiltration of primed T cells that subsequently attack the components of the myelin sheet. The myelin sheath is a specialized extension of the plasmatic membrane of oligodendrocytes in the CNS. It wraps around the nerve axon allowing fast conduction of action potential in a saltatory manner in nodes of Ranvier, (myelin gaps rich in sodium channels) while providing nutrition to the axon, in particular the high demanding mitochondrial activity (Iglesias-Rozas and Garrosa 2013). The structural organization of myelin sheaths is essential for neuronal function and the CNS-abundant myelin basic protein (MBP) is responsible for the structural organization of the multilayers of myelin (Boggs 2006).

In MS, the multiple focal areas of demyelination within the CNS are called plaques or lesions, and the development of inflammatory and myelin sheath lesions that appear disseminated throughout the white matter regions of the brain, optic nerve, spinal cord, and even throughout cortical grey matter might be found (Mey et al. 2023). Peripheral immune cells overcome the protection of the BBB and infiltrate the CNS parenchyma. The active leukocytes can enhance BBB permeability due to the secretion of pro-inflammatory cytokines that can directly disrupt the tight junction architecture or induce neuroglial cells to do so. Once the BBB is disrupted, leukocytes can migrate and initiate lesion development (Larochelle et al. 2011). Chronic inflammatory demyelination causes axonal transection and degeneration, and contrary to peripheral nerve injury, the axons in the CNS and spinal cord cannot regrow and regenerate (Dutta and Trapp 2010). Therefore, neurodegeneration associated with the loss of axons, dendrites, and neurons is the fundamental cause of permanent neurologic damage in MS patients (Mey et al. 2023), being MS progression a combination of two processes: demyelination with the malfunction of remyelination and increasing axonal damage with no recovery.

The demyelination process in MS is thought to start in the paranode (adjacent to the nodes of Ranvier) and extend to the juxtaparanodal domains. In the lesion regions, the highly organized membrane proteins disperse throughout the uncovered axon, including the sodium channels of the Ranvier nodes. The redistribution of sodium channels causes conduction blockage and calcium overload, leading to axonal damage. This process requires the recruitment of oligodendrocyte progenitor cells (OPCs) and their maturation in the demyelinated lesions to start the process of remyelination (Sommer et al. 2022). Mature oligodendrocytes might also aid in the process. However, this remyelination is not enough for a full recovery in MS patients due to the extension of the lesions, loss of OPCs with age, and the long period that it takes to fully recover the myelin sheath (Brown et al. 2014) when destruction is faster during this pathological process. Furthermore, OPCs are particularly vulnerable to oxidative stress since these cells have a higher rate of oxidative metabolism than adult oligodendrocytes. Moreover, OPCs have increased intracellular iron content that is needed for differentiation and myelination, additionally hindering the remyelination of axons (Spaas et al. 2021) and also contributing to exacerbating oxidative stress. The dysregulation of OPCs differentiation causes iron accumulation both in extracellular deposits and intracellular structures of different neuronal cell types (Williams et al. 2012). Iron accumulation can be directly neurotoxic or indirectly by inducing oxidative stress via the Fenton reaction and exacerbating inflammation (Kamma et al. 2022). Regarding inflammation, iron overload promotes the microglia activation into the pro-inflammatory phenotype (O’Loughlin et al. 2018).

**Fig. 4:**
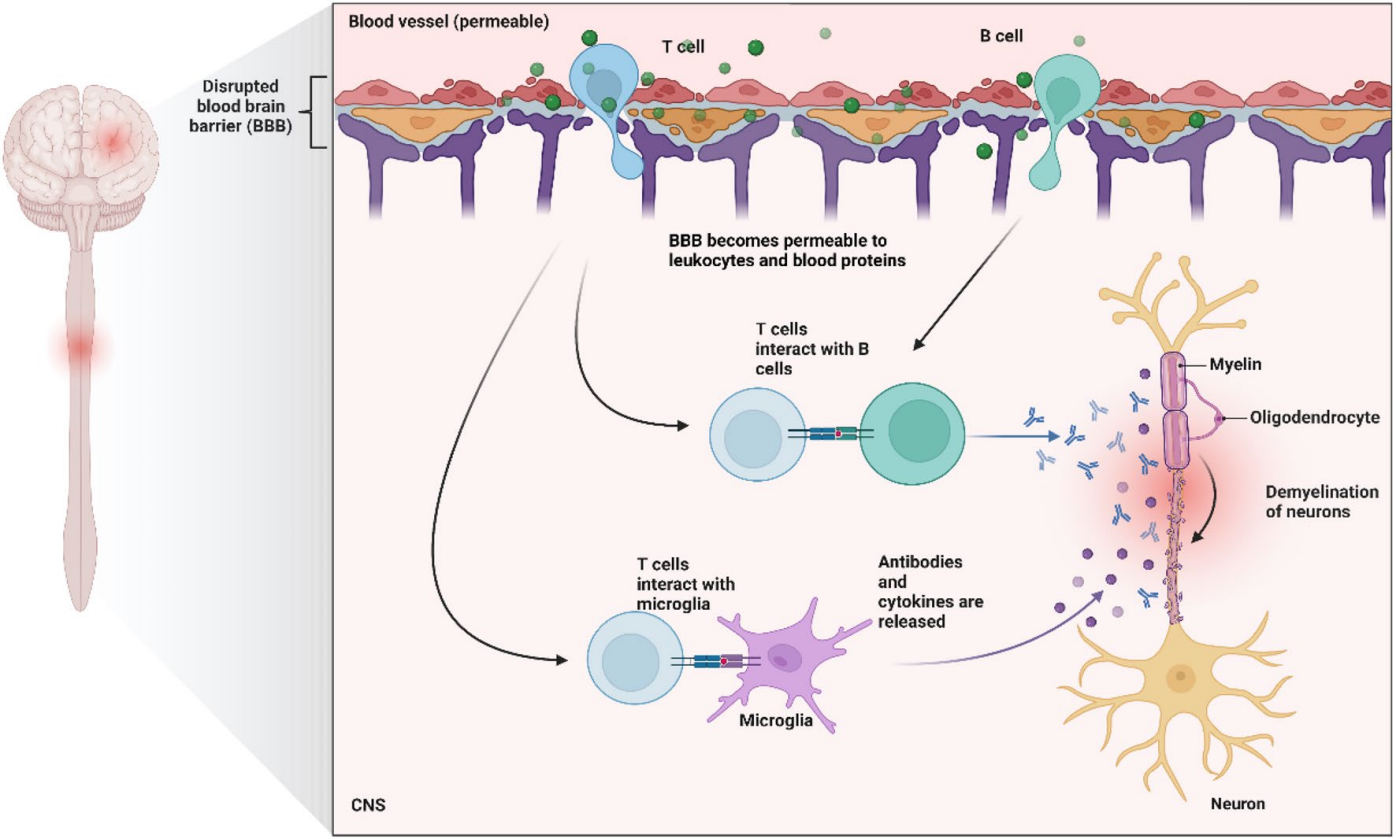
Schematic representation of the sequence of events in multiple sclerosis (MS). Active leukocytes and circulating pro-inflammatory cytokines (green dotes) disrupt the blood–brain barrier (BBB) and infiltrate the central nervous system (CNS) parenchyma. The primed T cells interact with B cells to produce antibodies against the components of the myelin sheet. Furthermore, T cells also interact with microglia to release antibodies and cytokines that further destroy the myelin sheet. Created with BioRender.com

Regarding the MS lesions caused by the above-mentioned events, the most common white matter lesions could be considered acute active plaques or chronic plaques (Popescu et al. 2013). Acute active plaques are characterized by hypercellular demyelinated areas infiltrated by macrophages containing myelin debris. Perivascular and parenchymal inflammatory infiltrates are composed mostly of T CD8^+^ cells, lesser T CD4^+^ cells, B cells, and plasma cells. Extensive astrogliosis is also observed in MS patients (Popescu and Lucchinetti 2012). Chronic plaques are characterized by completely demyelinated cores, with substantial loss of axons and oligodendrocytes, with myelin breakdown in the edges (Calvi et al. 2020). The border of the plaque has myelin-load macrophages and active microglia (with some cells containing myelin debris) (Popescu and Lucchinetti 2012). Chronic plaques can also be more inactive, with complete demyelinated centers, a considerable loss of axons and oligodendrocytes, astrogliosis, and minimal infiltration by macrophages, microglia, and lymphocytes (Popescu and Lucchinetti 2012). In fact, inflammation is consistently present in nearly all lesion types and disease stages of MS (Fig. 4).

Depending on the initial disease course, MS can be classified into clinical subtypes: relapsing–remitting MS and primary progressive MS (Nazeri et al. 2022). Relapsing–remitting MS is more common, accounting for 85–90% of the cases of MS, and it is defined by episodes of neurological dysfunction for at least 24 h without fever or infection (relapses) followed by periods of remission. Primary progressive MS affects 10 to 15% of the patients and it comes with a gradual increase in neurological impairment, without relapses (Mey et al. 2023). After the initial presenting symptoms, such as fatigue, vision problems, muscle weakness, spasms and stiffness, pain, and cognitive difficulties, the diagnosis is usually made by the observation of brain magnetic resonance imaging, which typically shows white matter lesions in particular areas (Brownlee et al. 2017).

MS is a prototypical disease of peripheral inflammation leading to neurodegeneration. As stated, this neurological disorder is not usually aging-related but is fully characterized as an immune disease, whose mechanisms will be compared with AD and PD in the discussion/conclusion section and for that reason chosen to be mentioned herein.

## Multiple sclerosis immunological signature

**Fig. 5:**
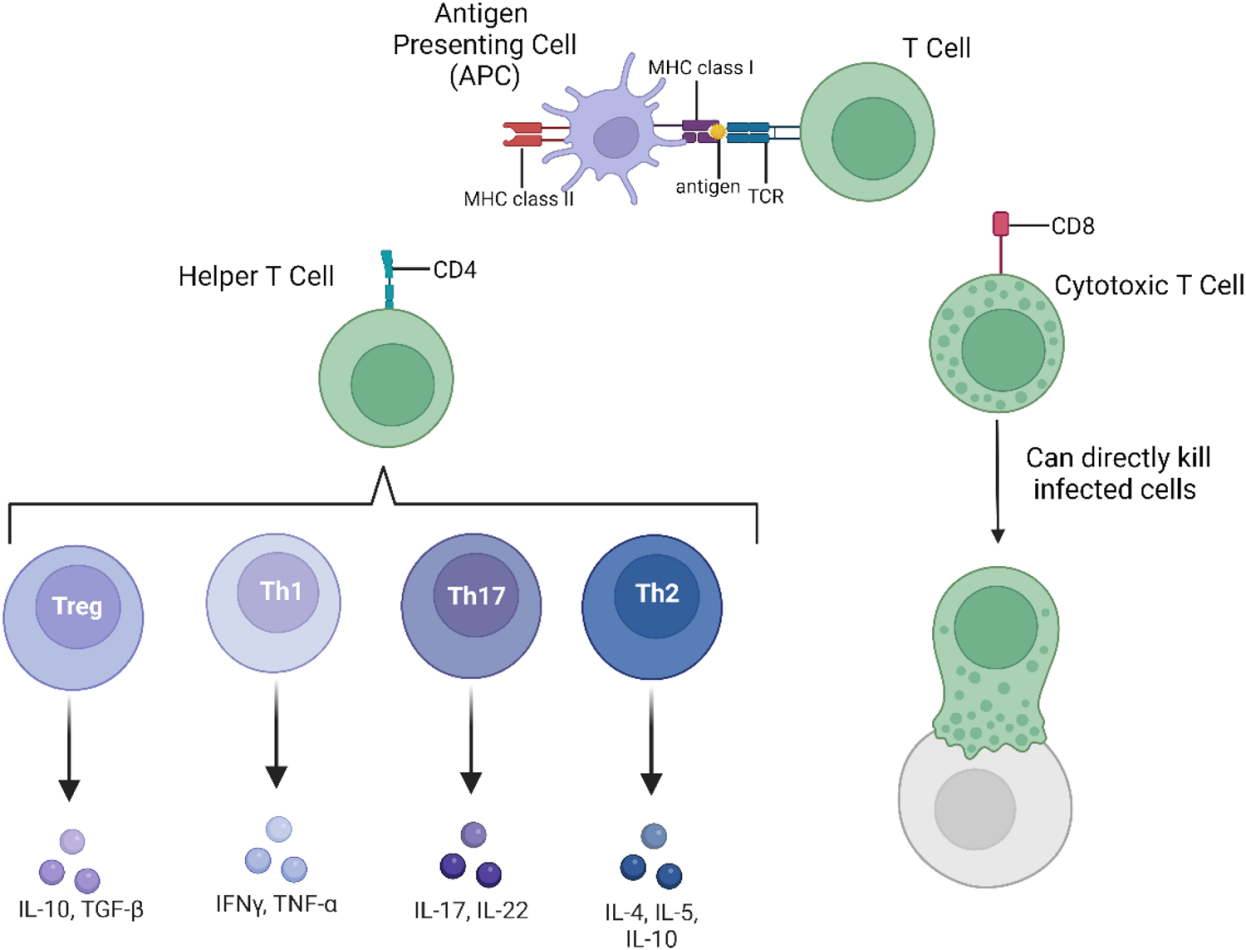
Schematic representation of the immune cells that mediate multiple sclerosis (MS). T cell receptors (TCR) can recognize antigens but do not bind directly, they require antigen-presenting cells (APC). APC express histocompatibility complex (MHC), of which, MHC class I presents antigens to cytotoxic T cells and MHC class II present to helper T cells. Furthermore, to make the connection between the TCR and MHC, the T cells express co-receptors. Usually, the cytotoxic T cells express the CD8 co-receptor, and the helper T cells express the CD4 co-receptor. The cytotoxic T cells can directly kill infected cells. The helper T cell can be divided into different phenotypes according to the specific pattern of cytokines secreted. Created with Biorender.com

MS immune-mediated chronic inflammation initiates in the periphery with failure in self-recognition by T cells. The antigens that trigger the immune response are not elucidated but some potential targets are myelin proteins and heat-shock proteins (Gonsette 2012). T cells can recognize non-self-particles (or antigens) due to surface T cell receptors (TCR). They can differentiate either into cytotoxic T cells that can directly kill infected cells or into helper T cells capable of activating other cells such as B cells and macrophages (Adams et al. 2020). The TCR cannot bind to antigens directly, as they require antigen-presenting cells (APCs), which break down the antigen into smaller peptides. On the surface of APCs, MHC molecules present the antigen to T cells: MHC class I presents the antigen to cytotoxic T cells and MHC class II presents the antigen to helper T cells. Furthermore, since the binding of the TCR to the MHC molecule that contains the antigen to be presented is unstable, T cells express co-receptors to aid the connection. Usually, helper T cells express the CD4 co-receptor and the cytotoxic T cells express the CD8^+^ co-receptor (Mørch et al. 2020). Additionally, naïve T cells (either matured CD4^+^ or CD8^+^ but not yet exposed to antigen), upon exposure to determined cytokines can differentiate into a T cell phenotype with associated production of specific cytokines (Kasper and Shoemaker 2010). T CD4^+^ cell phenotypes can be divided into 4 subsections: pro-inflammatory Th1 and Th17 cells, anti-inflammatory Th2, and regulatory T cells (Treg) (Sato et al. 2016). The aforementioned immune cells and their relationship are summarized in Fig. 5.

Regarding the autoimmune etiology of MS, for many years it was believed to be a helper T CD4-mediated disease. This theory was based on the observation that the transfer of myelin-specific T CD4^+^ cells to naïve animals caused autoimmune encephalomyelitis, which has been seen since then as an experimental model of MS with an inflammatory demyelinating ongoing process (Jones et al. 2016). Furthermore, regarding the aforementioned HLA genetic risk, the strongest linkage disequilibrium was found particularly in regions that encoded for the MHC class II (De Jager and Hafler 2010). For many years, Th1 cells were believed to be the primary drivers of the autoimmune process seen in MS. The cytokine IL-12 induces the phenotype Th1 and in turn, these cells secret pro-inflammatory cytokines including IFN-γ, TNF-α, and IL-2, reported to be elevated in serum samples of MS patients (Martins et al. 2011; Sato et al. 2016). The skewed Th1/Th2 balance was believed to be behind MS progression. On the other hand, Th2 cells produce cytokines such as IL-4, IL-10, or IL-5 linked to anti-inflammatory functions (Oreja-Guevara et al. 2012). MS patients with higher activity of Th2 cells usually have fewer relapsing episodes (Kallaur et al. 2013; Oreja-Guevara et al. 2012). Th1 and Th2 cytokines can cross-inhibit each other, and depending on the ratio, the progression or remission of the disease is triggered (Oreja-Guevara et al. 2012; Sato et al. 2016). In MS patients, pharmaceutical immunomodulation to increase Th2 results in beneficial effects on the course of the illness (Oreja-Guevara et al. 2012; Tselis et al. 2007). In addition, in SJL/J mice, where helper T cells were polarized to either Th1 or Th2 phenotypes, it was demonstrated that the Th1 phenotype induced autoimmune encephalomyelitis whereas the Th2 phenotype did not (Khoruts et al. 1995).

Conversely, the central role of Th1 cells in MS was challenged when inhibition of IL-12 was not efficacious in reducing MS lesions in phase II clinical trials (Segal et al. 2008). In addition, IL-12 knockout mice were not able to generate Th1 cells but were still susceptible to autoimmune encephalomyelitis. On the contrary, IL-23 deficient mice are resistant to experimental induction of autoimmune encephalomyelitis, suggesting an important role of this cytokine (Cua et al. 2003). IL-23 drives the differentiation of the Th17 population that secretes several pro-inflammatory cytokines including IL-17 and IL-22 (Jadidi‐Niaragh and Mirshafiey 2011). Considering this, it was observed that in MS patients, IL-17 levels in the CSF and peripheral blood mononuclear cells were elevated in comparison to healthy controls. Furthermore, post-mortem analysis of brain tissue from MS patients revealed increased expression and production of IL-17 mainly in active lesion sites. Besides T cells, also astrocytes and oligodendrocytes produce this cytokine. Regarding T cells, high density of both T CD4^+^ and T CD8^+^ cells were found in active lesion sites and producing IL-17. On the other hand, no Treg cells were detected, suggesting no T cell inhibition in the CNS (Tzartos et al. 2008). Treg cells can control disease and prevent its progression since they can suppress T cell activation by inhibiting its proliferation and cytokine release (Kondĕlková et al. 2010). In MS patients, Treg cells are reported to have abnormal protein expression levels of FOXP3, a protein essential for the acquisition of suppressive functions (Huan et al. 2005). The immune system is characterized by its redundancy and complex interlinks and associations between its components, therefore, while autoimmunity might be more dependent on one or more components, it seems clear that several immunological checkpoints fail in MS.

The singular impact of T CD4 cells in MS progression was further questioned when depletion of this cell subtype did not show improvement in MS outcome in a randomized, double-blind, placebo-controlled exploratory trial (van Oosten et al. 1997). Besides the clear involvement of T CD4^+^ cells, T CD8^+^ cells are not harmless standbys in MS. When target therapy was used for the ablation of T cells, a significant reduction in MS relapse was observed (Coles et al. 2008). In active MS lesions, T CD8^+^ cells can outnumber T CD4^+^ cells independently of the MS subtype, time, and speed of disease progression (Booss et al. 1983; Ramagopalan et al. 2010). The initial view was that T CD8^+^ cells would suppress other T cells via direct cytotoxic activity and, in that way, regulate and block MS progression (Antel et al. 1984). However, increasing data suggest that this T cell subtype is a major contributor to the immunopathogenesis of MS. Post-mortem brain analysis of MS patients exposed that neurons, axons, astrocytes, and oligodendrocytes present upregulated expression of MCH class I in comparison to healthy controls. Therefore, neuronal and glial cells could be targeted by T CD8^+^ cells, suggesting their involvement in the destruction of myelin and axons (Höftberger et al. 2004). In general, activated T CD8^+^ cells kill target cells by placing granzymes into the cytosol of targeted cells and by the direct release of vesicles containing enzymes and perforin (Osińska et al. 2014). Recently, considering the growing interest in the role of IL-17 in MS, a subtype of T CD8^+^ cell that secretes IL-17 was found to be particularly active in inflammatory lesions (Tzartos et al. 2008). Furthermore, T CD8^+^ IL-17 secreting cells were found to increase in the CSF of early-stage MS patients but not in the peripheral blood, indicating that the inflammatory microenvironment of the brain might trigger the clonal expansion of these cells and the production of IL-17 (Acosta-Rodriguez et al. 2007). This notion is supported by the presence of IL-23, a cytokine that induces the IL-17-secreting phenotype in T CD8^+^ and that is produced by macrophages and dendritic cells located in MS lesions (Beriou et al. 2009). In addition, IL-6 and IL-1β also differentiate naïve T cells into IL-17-secreting cells (Annibali et al. 2011).

Besides the involvement of several subtypes of T cells, B cells may also contribute to MS pathogenesis in several ways, namely by regulating and secreting cytokines, promoting antigen presentation for activation of T cells, and by being the precursors of antibody-secreting plasma cells (Lehmann-Horn et al. 2013). Furthermore, the intrathecal presence of antibody-secreting plasma cells, B cells in MS brain plaques, meningeal clusters of B cells, and oligoclonal bands secreted by B cells were found in CSF of MS patients (Nissimov et al. 2020; Siritho and Freedman 2009). These observations further demonstrate the importance of B cells in MS progression. In addition, memory B cells express high levels of the surface protein CD20 (a prototypical marker of B cells), and the anti-CD20 therapies developed since then abrogate these cells and have proved to be very effective in improving and/or attenuating MS symptoms (Nissimov et al. 2020). Those clinical effects seem to be related to the lack of the antigen-presenting function that drives T cell activation (Lehmann-Horn et al. 2013). Moreover, a few years ago a study found that a small subset of human T cells that express low levels of CD20 (Hultin et al. 1993) was found but only recently more attention has been paid to this subpopulation due to the effectiveness of anti-CD20 therapies. The T CD20-expressing cells have a Th1 pro-inflammatory profile with great proliferative capacity against CNS antigens (von Essen et al. 2018). This cell type is increased in the blood and CSF of MS patients, and its concentration in the CSF positively correlates with the severity of the disease (von Essen et al. 2018). While there is evidence for the involvement of T CD20-expressing cells in MS, little is known about how they acquire this surface protein. Promising research has demonstrated that this subtype requires interaction with B cells expressing CD20 to develop this surface protein, possibly via trogocytosis (Ochs et al. 2022).

Naïve T cells are activated in MS, but the source antigen is still unknown. There is some evidence for MBP as a primary antigen source (Martinsen and Kursula 2022) but T cell priming due to peripheral inflammation (e.g., pathogens or environmental exposures) has also been suggested (Salou et al. 2015). T cells differentiate into mature effector T CD4^+^ cell subtypes (Th1, Th2, Th17, and Treg), depending on the cytokine secreted by the APC. The data suggest that MS progression is driven both by Th1 and Th17 subtypes via different mechanisms. The auto-aggressive T cells interact with the BBB and cross it, infiltrating into the brain parenchyma. Once in the brain, these cells interact not only with the myelin but also with other resident immune cells, further increasing brain damage (Chastain et al. 1812). Additionally, it appears that T CD4^+^ cells initiate the autoimmune process and T CD8^+^ cells carry the demyelination lesion (Höftberger et al. 2004).

## Integration of inflammation in neurological diseases

Due to the natural loss of functions in many biological systems, older individuals require special attention and care. Neurologic disorders are the main disability factors, with a great impact on the quality of life in older adults. Moreover, with advanced age, the pro-inflammatory status of the individual increases, being that low-grade chronic inflammation contributes to the development of many diseases and accelerated aging. While for many years it was believed that the CNS was immune-privileged, research is continuously proving that there is extensive communication and mutual influence between the CNS and the immune system. Therefore, one cannot ignore the contribution of chronic low-grade inflammation to the development of neurological disorders.

The most common progressive neurological disorders are AD and PD. They present very distinguishable characteristics, but at the core, both diseases present very similar features, such as misfolded proteins that form aggregates and disrupt the CNS. While AD and PD are neurological diseases deeply linked to aging, MS is not considered a classical age-related neurodegenerative disease. Nevertheless, MS shares some features and mechanisms of aging-related neurodegeneration observed in AD and PD. MS often appears in mid-life and progresses over time while in AD and PD the diagnosis happens later in life. However, the first signs might start decades earlier being often overlooked. As described previously, the course of the three diseases starts with peripheral immune dysregulation, followed by stimulation of neuroinflammation with consequential neuronal damage, and degeneration that causes cognitive and motor impairment. AD and PD are characterized by extensive protein misfolding, and more recent studies have also found protein aggregates of Aβ and Tau in the brain and CSF of MS patients (David and Tayebi 2014; Lassmann 2011), further confirming the share mechanisms of progression of the disease.

Research presented here demonstrates how the increase in peripheral cytokines causes a downstream of events leading to neuroinflammation, protein misfolding, and neuronal degeneration with consequential cognitive impairment. Furthermore, when the inflammation is tackled at the very beginning, for example by the prolonged use of non-steroidal anti-inflammatory drugs, there was a reduction in the risk of developing both AD and PD. This further suggests that inflammation is involved in the very early start of the disease and once it begins there is little to no solution.

The difficulty in controlling unresolved inflammation is mainly due to the redundancy and multiple effectors of the immune system that can amplify the signal of various inflammatory factors. This fact was observed mainly in the prototypical inflammatory neurodegenerative disorder of MS. Including MS in a study of age-related neurodegeneration and aging is essential to understand the inflammatory triggers that could contribute to immune dysregulation, also observed in AD and PD, and to identify potential therapeutic targets. The research on this disease demonstrated that, while it seems that some immune cells have a more prominent role, their ablation does not resolve the inflammatory process. The etiology of MS is not known, but it seems to be related to exposure to substances that trigger the immune system and cause an increase in inflammatory factors, which can be shared with PD or AD characteristically age-related disorders. Nonetheless, the role of inflammation in neurological diseases or at least some niches is certain, and that notion can change the perspective, treatment, and prevention of those illnesses.

While this review is focused on the increase of inflammation with advanced age and on older individuals, it is important to emphasize that neuroinflammation consequences can be deleterious at any age. In fact, recent studies have highlighted the pathogenic role of neuroinflammation in several neurological disorders that happen early in life such as schizophrenia, autism spectrum disorders, bipolar disorder, depression and obsessive–compulsive disorder (Jacqueline et al. 2017; Najjar et al. 2013). The incidence of all these diseases have been significantly increasing (Tondo et al. 2021) and they are very debilitating. If the root of these diseases is, in fact, inflammation, does it still make sense to try to prescribe the same treatments that fail numerous patients time and time again? Moreover, can inflammatory regulatory aspects be triggered in these patients and can chemokines and cytokines work as early diagnosis biomarkers? More research is needed to understand how inflammation affects mental function and most definitely perspective on inflammation as a general and not specific aspect of neurological diseases needs to be challenged.

## Data Availability

Not applicable.

## Abbreviations

AD: Alzheimer’s disease
APCs: Antigen-presenting cells
ApoE: Apolipoprotein E
APOE-e4: Apolipoprotein-e4
APP: Amyloid precursor protein
Aβ: Amyloid-β peptides
BBB: Blood–brain barrier
CNS: Central nervous system
CSF: Cerebrospinal fluid
GFAP: Glial fibrillary acidic protein
HLA: Human leukocyte antigen
IFN-γ: Interferon gamma
IL: Interleukin
JAK: Janus kinase
MBP: Myelin basic protein
MHC: Major histocompatibility complex
MPTP: 1-Methyl-4-phenyl-1,2,3,6-tetrahydropyridine
MS: Multiple sclerosis
NFTs: Neurofibrillary tangles
NF-κB: Nuclear factor kappa B
OPCs: Oligodendrocyte progenitor cells
PD: Parkinson’s disease
STAT: Signal transducer and activator of transcription
TCR: T cell receptor
TGF-β: Transforming growth factor beta
TNF: Tumor necrosis factor
Treg: Regulatory T cells
α-2 M: Alpha-2 macroglobulin
